# Development of a high-throughput colorimetric Zika virus infection assay

**DOI:** 10.1007/s00430-017-0493-2

**Published:** 2017-02-07

**Authors:** Janis A. Müller, Mirja Harms, Axel Schubert, Benjamin Mayer, Stephanie Jansen, Jean-Philippe Herbeuval, Detlef Michel, Thomas Mertens, Olli Vapalahti, Jonas Schmidt-Chanasit, Jan Münch

**Affiliations:** 1 Institute of Molecular Virology, Ulm University Medical Center, Ulm, Germany; 2 Institute of Virology, Ulm University Medical Center, Ulm, Germany; 3 Institute of Epidemiology and Medical Biometry, Ulm University, Ulm, Germany; 4 Bernhard Nocht Institute for Tropical Medicine, World Health Organization Collaborating Centre for Arbovirus and Hemorrhagic Fever Reference and Research, Hamburg, Germany; 5 German Centre for Infection Research, Partner sites Hamburg-Luebeck-Borstel, Hamburg, Germany; 6 CBMIT CNRS UMR-8601, Université Paris Descartes, CICB, Paris, France; 7 Departments of Virology and Immunology, and Veterinary Biosciences, University of Helsinki and Helsinki University Hospital, Helsinki, Finland

**Keywords:** MTT, ZIKV, Zika virus, PRNT, Screening

## Abstract

Zika virus (ZIKV) is an emerging pathogen that causes congenital infections which may result in birth defects, such as microcephaly. Currently, no approved treatment or vaccination is available. ZIKV can be readily detected in cell culture where virally infected cells are normally stained by specific antibodies. As ZIKV regularly causes a cytopathic effect, we were wondering whether this viral property can be used to quantitatively determine viral infectivity. We here describe the use of an 3-[4,5-dimethyl-2-thiazolyl]-2,5-diphenyl-2H-tetrazolium bromide-(MTT)-based cell viability assay that allows to determine ZIKV-induced cell death. We show that this colorimetric assay quantifies ZIKV infection over a broad range of viral dilutions in both monkey and human cells. It allows to determine inhibitory activities of antivirals that block ZIKV or to define the neutralizing antibody titers of ZIKV antisera. This MTT-based ZIKV detection assay can be evaluated by naked eye or computational tools, has a broad linear range, does not require large equipment or costly reagents, and thus represents a promising alternative to antibody-based assays, in particular in resource-poor settings. We propose to use this simple, fast, and cheap method for quantification of ZIKV neutralizing antibodies and testing of antiviral compounds.

**Electronic supplementary material:**

The online version of this article (doi:10.1007/s00430-017-0493-2) contains supplementary material, which is available to authorized users.

## Introduction

Since the first recognized large outbreak of ZIKV in Micronesia in 2007, the virus spread rapidly and has now caused a major epidemic with an estimated number of more than 1 million infected individuals in Brazil [1–3]. Although most infections are subclinical or mild, congenital ZIKV infection may result in severe birth defects, such as microcephaly [4]. In addition, ZIKV infection is suspected to be associated with Guillain–Barre syndrome in adults [5]. Today, neither a protective vaccine nor a specific antiviral therapy is available to prevent or cure ZIKV infections. The virus is mainly transmitted by mosquitos, but congenital, perinatal, and sexual modes of transmission have also been described [6]. ZIKV is a flavivirus that has a positive-sense single-stranded RNA genome and is surrounded by a lipid bilayer which makes it susceptible to, e.g., alcoholic disinfectants [7].

ZIKV diagnosis is based on direct amplification of viral RNA from patient material using “in house” or commercially available RT-PCR assays [8–10]. Serological binding assays have also been approved but are often restricted to reference laboratories and have limitations, because ZIKV IgM and IgG antibodies are cross-reactive with other flaviviruses. Particularly, dengue virus circulating in the same areas, and the prodromi and acute clinical symptoms are similar as in ZIKV infection [10–13]. Thus, positive serological tests should be confirmed by virus neutralization assays in cell culture. One widely used assay is the plaque reduction neutralization test (PRNT) which is based on the capability of flaviviruses to cause formation of plaques in cell monolayers [10, 14]. This cytopathic effect (CPE) can be observed directly in cell culture or after live cell staining. Alternatively, infected cells can also be visualized by immunostaining with virus-specific antisera or monoclonal antibodies. However, regardless of the method of visualization, in PRNT, plaques are usually counted manually. Quantification of infected cells is also routinely performed by immunostaining to study, e.g., viral tropism or the effect of antiviral compounds [15, 16]. Here, infected cells are quantified by detection of viral antigen by flow cytometry or ELISA [15, 17]. These assays are time-consuming and require specific equipment like a flow cytometer or microplate readers.

In this study, we describe a simple, fast, cheap, and robust assay to measure ZIKV infectivity and its inhibition by antisera or antivirals. The assay is based on the colorimetric detection of live cells using the tetrazolium salt MTT. Live cells reduce the yellow MTT solution by the NAD(P)H-dependent oxidoreductase system resulting in the formation of insoluble purple formazan crystals [18, 19]. Vice versa, dead cells or cells with impaired metabolism do not reduce MTT. Since ZIKV is able to cause cytopathic effects in cell culture, infected cells die which results in a decreasing production of formazan crystals. We here show that the MTT-based cell viability assay allows quantification of ZIKV infectivity and its inhibition by interferon or patient sera. The assay can be evaluated by naked eye, has a broad linear range, does not require expensive equipment or costly reagents, and thus represents an interesting alternative for ZIKV detection, in particular in resource-poor environment.

## Materials and methods

### Cells, viruses, and reagents

Vero E6 cells (ATCC^®^ CRL-1586™), used for propagation and infection with ZIKV, were grown in Dulbecco’s modified Eagle’s medium (DMEM) supplemented with 2.5% heat-inactivated fetal calf serum, 2 mM L-glutamine, 100 units/ml penicillin, 100 µg/ml streptomycin, 1 mM sodium pyruvate, and non-essential amino acids (Sigma #M7145) at 37 °C in a 5% CO_2_ humidified incubator. Human osteosarcoma (HOS) cells (NIH AIDS Reagent Program #3942) were grown in DMEM supplemented with 10% heat-inactivated fetal calf serum, 2 mM L-glutamine, 100 units/ml penicillin, and 100 µg/ml streptomycin. ZIKV strain MR766 was isolated in 1947 from a sentinel rhesus macaque. FB-GWUH-2016 is a ZIKV strain that was isolated in 2016 from a fetal brain with severe abnormalities [20]. The FB-GWUH-2016 stock used in this study was passaged two times on Vero E6 cells. Recombinant interferon-α (IFN-α) was purchased from pbl assay science (#11101-1).

### Virus propagation

Virus was propagated by inoculation of 70% confluent Vero E6 cells in T175 cell culture flasks for 2 h in 5 ml medium. Subsequently, 40 ml fresh medium was added and the cells cultured for 3 to 5 days. Cytopathic effect (CPE) was monitored by light microscopy and virus was harvested when 70% of the cells detached due to CPE. Supernatant was taken, centrifuged for 3 min at 1,300 rpm, before virus stocks were aliquoted and stored at −80 °C.

### MTT (3-[4,5-dimethyl-2-thiazolyl]-2,5-diphenyl-2H-tetrazolium bromide) assay

After incubation of Vero E6 or HOS cells with or without virus for the length of the according experiment, 20 µl of MTT solution (5 mg/ml in PBS) was added to 200 µl cells. Following a 3 h incubation time at 37 °C, the cell-free supernatant was discarded and formazan crystals were dissolved in 100 µl of a 1:2 mixture of dimethyl sulfoxide and ethanol. Absorption was measured at 490 nm and baseline corrected at 650 nm using a VMax Kinetic ELISA microplate reader (Molecular Devices). To determine infection rates, sample values were subtracted from untreated control and untreated control set to 100%. Error bars are standard deviations of triplicates.

### CellTiter-Glo^®^ Luminescent Cell Viability Assay

CellTiter-Glo^®^ Luminescent Cell Viability Assay (Promega #G7571) was performed according to the manufacturer’s instructions. Briefly, medium was removed from the cells, and 50 µl PBS and 50 µl of reagent were added. After a 10 min incubation, luminescence was measured in a Orion II Microplate Luminometer (Titertek Berthold).

### Cell-based Zika virus immunodetection assay

Immunodetection of ZIKV-infected cells was done as described [17]. Cells were rinsed with PBS, fixed for 20 min at room temperature with 4% paraformaldehyde, permeabilized with cold methanol for 5 min at 4 °C, and washed with PBS. Next, cells were incubated with anti-flavivirus mouse antibodies 4G2 from B lymphocyte hybridoma cells (ATCC^®^ HB-112^™^) (1:100) in PBS containing 10% (v/v) FCS and 0.3% (v/v) Tween20 for 1 h at 37 °C. Following three times of washing with PBS containing 0.3% (v/v) Tween20, cells were incubated with a HRP-coupled anti-mouse antibody (1:20,000) (Thermo Fisher Scientific #A16066) for 1 h at 37 °C. Next, cells were washed four times and TMB substrate was added. After 5 min of room temperature incubation, reaction was stopped with 0.5 M sulfuric acid. Absorption was measured at 450 nm and baseline corrected at 650 nm using an ELISA microplate reader. Error bars are standard deviations of triplicates.

### Patient sera

ZIKV-specific antisera were obtained from the reference collection of the WHO Collaborating Centre for Arbovirus and Hemorrhagic Fever Reference and Research, Hamburg, Germany [21]. Anti-ZIKV IgG and IgM titers were determined by indirect immunofluorescence assay (IIFA) using Vero E6 cells and ZIKV strain MR766. In brief, infected cells were spread onto slides, air dried, and fixed in acetone. Serum samples were serially diluted in phosphate-buffered saline (PBS) starting with an initial dilution of 1:10, added to the cells, and incubated for 90 min at 37 °C. After washing with PBS, slides were incubated with fluoresceine isothiocyanate-labeled rabbit anti-human IgG and IgM antibodies at 37 °C for 25 min. IgG titers or IgM titers of 1:20 or more were considered positive.

Control antisera and antisera containing antibodies against DENV and TBEV were derived from the Institute of Virology, Ulm. Qualitative detection of antibodies against DENV and TBEV was performed by ELISA according to the manufacturer’s instructions (Dengue Virus IgG DxSelect™ (EL1500G) Focus Diagnostics; Dengue IgM capture ELISA (01PE20) Panbio; TBE IgG/IgM ELISA (EC117.00) Sekisui Diagnostics/Virotech). All sera were tested for cytotoxicity on Vero cells and were not toxic at concentrations of 1% (v/v).

### MTT based neutralization assay

6,000 Vero E6 cells were seeded per 96 well in 100 µl medium and cultured overnight. For infection, 10^6^ TCID_50_/ml of ZIKV virus was incubated at room temperature with PBS or with 0.08, 0.16, 0.31, 0.63, 1.25, 2.50, 5, and 10% (v/v) heat-inactivated serum samples for 90 min. Cell medium was replaced by 180 µl fresh medium, and 20 µl of the ZIKV-serum mix was added in triplicates. 4 day post-inoculation, infection was measured by MTT assay.

### PRNT assay

6,000 Vero E6 cells were seeded in 96-well plates the day before the experiment. The patient sera/antisera were titrated twofold in medium from 1:20 to 1:163,840 and incubated with 100 PFU/ml ZIKV MR766 for 60 min at 37 °C. Next, 100 µl of the ZIKV/serum mix was added to 100 µl cells in triplicates and cells were monitored microscopically. Five day post-inoculation, cytopathic effects were best visible, and the lowest titer that inhibited ZIKV infection was determined.

### Statistics

Z’ factor was calculated as described [22, 23] and MTT assay-derived EC_50_s and titers were determined by GraphPad Prism (GraphPad Software, Inc.)

## Results

To study whether the MTT-based cell viability assay allows quantification of ZIKV infectivity, we monitored the formation of the virus-induced cytopathic effect (CPE) in Vero E6 cells that were infected with serial tenfold dilutions of the MR766 strain [24]. The applied ZIKV stock had an infectious dose of 1.6 × 10^7^ TCID_50_/ml and a genome copy number of ~1 × 10^10^ /ml [7]. Cells were monitored microscopically, and already after 2 days, plaques and detached cells were observed in wells infected with the two highest virus concentrations (not shown). After 4 days, a pronounced CPE was observed in wells infected with 10^1^ to 10^5^-fold dilutions of the virus, and at the 10^6^-dilution still single plaques could be detected (examples shown in Fig. S1A). Next, 20 µl of a MTT solution was added to cells at day 4 post-inoculation. Following a 3 h incubation at 37 °C, the supernatants which contain detached and dead cells were discarded, and the formazan crystals formed by adherent live cells were solubilized in 100 µl of a 1:2 mixture of dimethyl sulfoxide and ethanol, which also inactivates virus [7]. Visual examination of the microtiter plate showed that wells containing uninfected cells or cells that were inoculated with viral dilutions exceeding 10^5^ contained a dark purple solution (Fig. S1B). In contrast, wells that were infected with a dilution of the virus stock that causes a pronounced CPE (Fig. S1A) contained a clear transparent solution (Fig. S1B). Inoculation with 10^2^–10^5^ dilutions of the virus resulted in a gradually decreased plaque formation (Fig. S1A) that correlated with increased color intensity (Fig. S1B). Thus, the development of formazan crystals in live cells allows for an indirect detection of the ZIKV-infected cells.

**Fig. 1:**
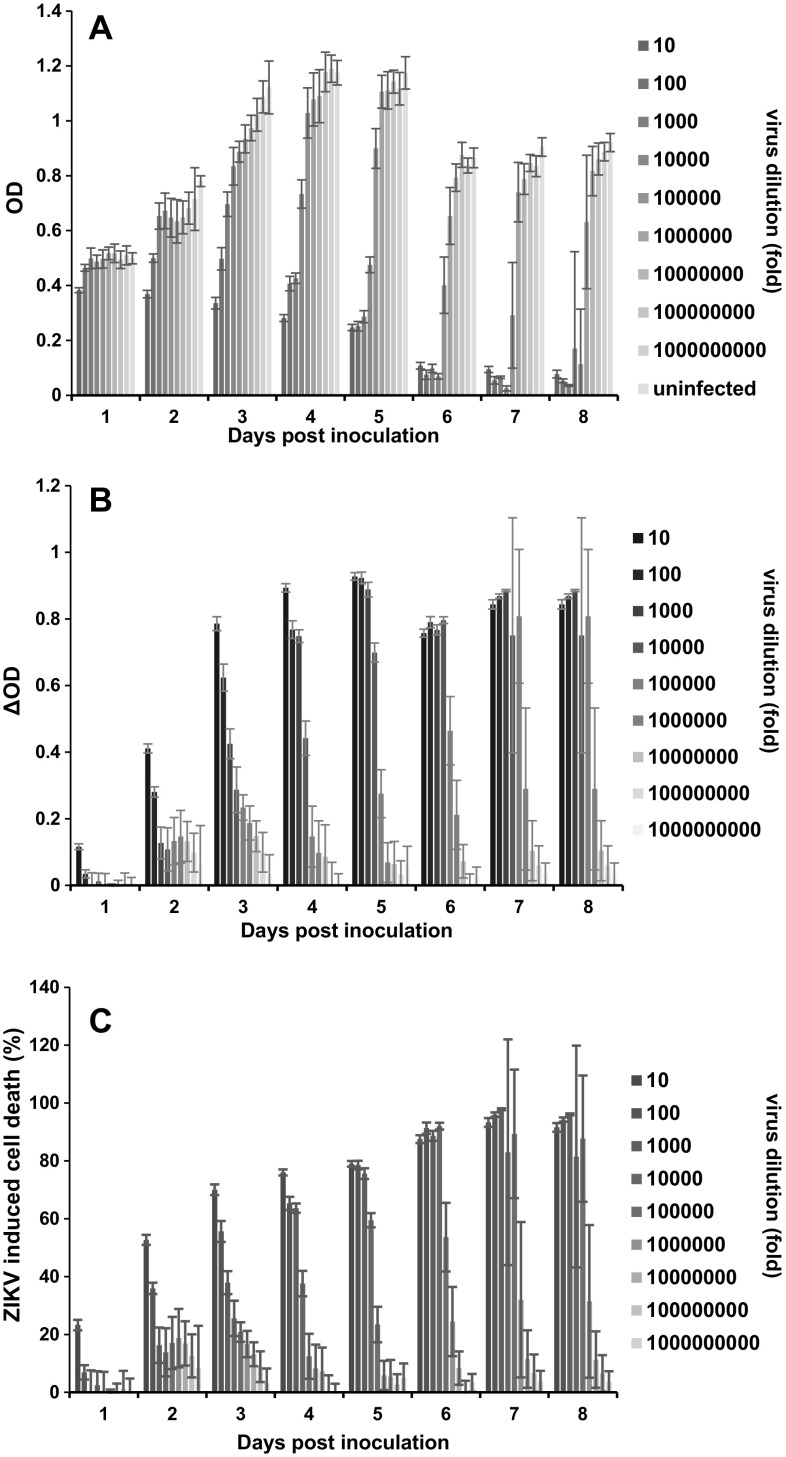
Representative results of the MTT-based assay to quantify ZIKV infection in Vero E6 cells. 6 × 10^3^ Vero E6 cells were seeded in 96-well plates. The next day, 20 µl of serial tenfold dilutions of the ZIKV strain MR766 were added. After 1–8 days, cells were monitored microscopically and washed once with medium to remove detached virally infected cells. Thereafter, 20 µl of MTT was added to remaining adherent cells in 200 µl. After 3 h, formazan crystals were dissolved in DMSO/ethanol and absorbance of the colored solution at 490 nm was quantified by a spectrophotometer. **a** Raw data obtained from live cells (optical densities; ODs) derived from triplicate infections ± standard deviation. **b** ODs derived from live cells in infected cell culture were subtracted from ODs obtained from uninfected control cells. The resulting ΔODs numbers provide an indirect value for all cells that detached due to virus infection. **c** Percentage of ZIKV-induced cell death was calculated by normalizing the ΔOD values to the OD value of the uninfected control

We next quantified formazan formation in cell cultures that were infected for 1–8 days with serial dilutions of the ZIKVMR766 stock. Color development was determined by recording the absorbance at 490 nm (OD, optical density) using an ELISA microplate reader (Fig. 1a). At day 1, only the highest virus dose caused a slight reduction of the OD, as compared to the uninfected control (Fig. 1a). At days 2 to 8, the ODs increased with increasing viral dilutions. (Fig. 1a). We next calculated the Z’ factor as a measure of statistical effect size which is used to validate the quality of high-throughput assays [22]. The Z’ factor was, on average, 0.77 demonstrating an excellent assay performance. The best dose-dependent correlation was observed at day 3 (*R* ^2^ = 0.90) (Fig. 1a and Fig. S2). Microscopic evaluation of cells infected with viral dilutions of 10^1^ to 10^4^ revealed pronounced plaque formation at day 6 to 8 (not shown), and consequently, OD values close to zero (Fig. 1a). Thus, quantification of live cells by MTT assay is an indirect measurement for ZIKV infectivity. Due to ongoing cell growth, ODs of uninfected cells increased from day 1 to 5 before contact inhibition occurred. At days 6 to 8, ODs slightly decreased probably due to a reduced metabolic activity of cells that were grown to high density and exhaustion of nutrients in the medium (Fig. 1a). To determine the fraction of cells that were killed by the virus, ODs obtained from infected wells were subtracted from ODs observed from the uninfected control wells (Fig. 1b). The resulting delta OD (ΔOD) values were then used to calculate the percentage of dead cells for each sample (Fig. 1c). This evaluation illustrates an increase in the percentage of dead cells with increasing ZIKV input and the time of infection (Fig. 1c).

The Vero E6 kidney epithelial cells extracted from African green monkey are commonly used in ZIKV research, even they are not of human origin. To evaluate whether the MTT-based assay allows ZIKV detection in human cells, human osteosarcoma (HOS) cells were inoculated with serial dilutions of the ZIKV MR766 stock. The virus was cytopathogenic at days 3 and 4 post-infection which allows the quantification of remaining life cells by MTT assay (Fig. S3). As previously shown for Vero cells, the ODs inversely correlated with the amount of input virus (Fig. S3A and S3B), and allowed to calculate the percentage of cells that were killed by the virus (Fig. S3C). Thus, the MTT-based ZIKV CPE reduction assay can be easily adapted to human cells.

**Fig. 2:**
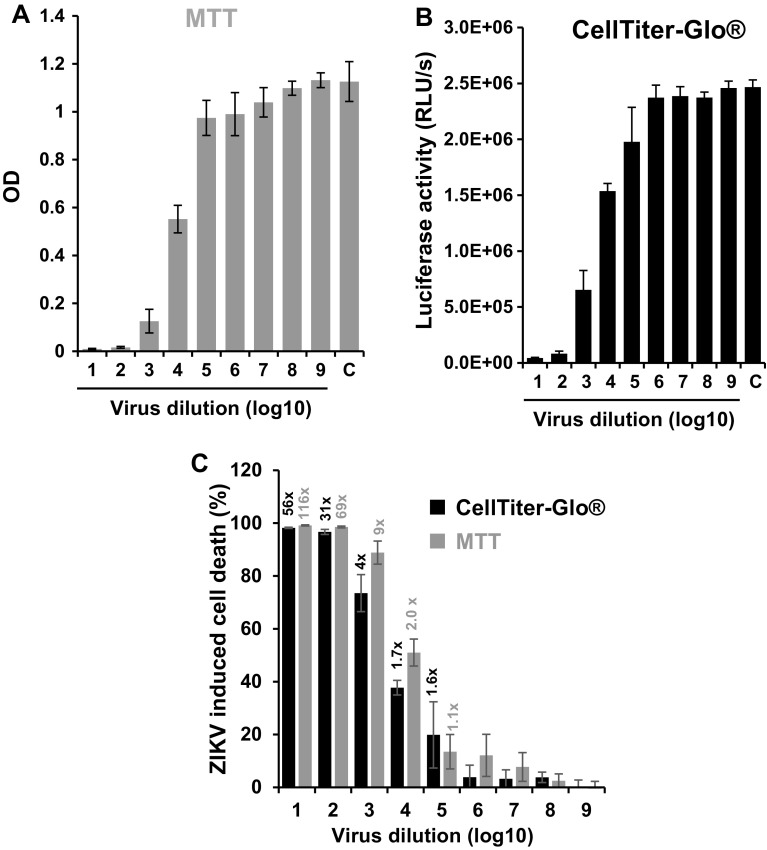
Comparison of the colorimetric MTT test with the enzyme-linked CellTiter-Glo^®^ Luminescent Cell Viability Assay to quantify ZIKV infectivity. Vero E6 cells were infected with serial 10-fold dilutions of ZIKV MR766 and assayed 4 day post-infection using either **a** MTT assay or **b** the CellTiter-Glo® Luminescent Cell Viability Assay. Shown are mean OD values (**a**) or luciferase activities (**b**) derived from triple biological replicates ± standard deviation. **c** Direct comparison of both cell viability assays. The percentage of ZIKV-induced dead cells was calculated as described above. The *numbers* above the *columns* give the signal-to-noise ratios, i.e., the quotient of infected versus uninfected cells. *C* uninfected control

We next compared the sensitivity of the MTT-based cell viability assay with the one of the CellTiter-Glo^®^ assays that determines intracellular levels of ATP by a luminescence-based reaction. The same assay principle has recently been used to screen for ZIKV inhibitors [25]. Here, we titrated ZIKV MR766 on Vero E6 cells and determined cell viabilities 4 days later by MTT (Fig. 2a) or CellTiter-Glo^®^ assay (Fig. 2b). Viral dilutions of 10^5^ or higher did not result in reduced OD values (Fig. 2a) or luciferase activities (Fig. 2b). Infection with 10^1^ and 10^2^ dilutions of the virus caused pronounced cell death as observed by microscopy (data not shown), and consequently, only very low levels of formazan production (Fig. 2a) or luciferase activity (Fig. 2b) were detected. Reduced viability rates were observed in wells infected with 10^3^ to 10^4^ dilutions of the virus (Fig. 2a, b). To directly compare the sensitivity of both assays, we calculated the percentage of dead cells (Fig. 2c) as described above (Fig. 1c). As expected, both assays produced similar results (Fig. 2c). The signal-to-basal ratios of the MTT-based cell viability assay were approximately twofold higher than that of the CellTiter-Glo^®^ assay (Fig. 2c) suggesting slightly higher sensitivity of the colorimetric assay. However, the Z’ factor of the luminescence-based assays was 0.91 and thus slightly higher than the Z’ factor for the MTT assays (0.77).

**Fig. 3:**
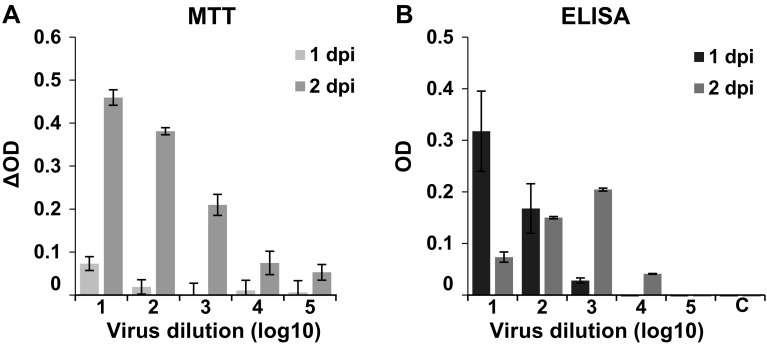
Comparison of the MTT- and a ZIKV immunodetection method. Vero E6 cells were infected with tenfold dilutions of ZIKV and after 1 or 2 days, viral infectivity was determined by **a** MTT assay (for better comparison, the ΔOD values are shown), or **b** immunodetection with the ZIKV antibody 4G2. For this, cells were fixed, incubated with 4G2, washed, and then incubated with a secondary HRP-coupled antibody. After additional washing steps, TMB substrate was added and color development quantified by ELISA plate reader. All values represent mean values ± standard deviation. *C* uninfected control

We next compared MTT-based ZIKV detection with a widely used immunofluorescence assay that utilized the pan anti-flavivirus mouse antibody 4G2 that binds ZIKV E protein [15, 26, 27]. Vero E6 cells were infected with the ZIKV MR766 strain and the MTT-based cell viability assay was performed after 1 and 2 days, and evaluated as described above (Fig. 3a). In parallel, cells were fixed, treated with the 4G2 antibody, washed, exposed to a secondary HRP-coupled anti-mouse antibody, and washed again, before TMB substrate was added. Color development was stopped with sulfuric acid and ODs measured at 450 nm (Fig. 3b). As expected from data shown in Fig. 1, the ΔOD values measured by MTT assay increased from day 1 to 2 due to virus-induced CPE, and gradually declined with decreasing amounts of input virus (Fig. 3a). When using the antibody-based approach, the highest OD was obtained at day 1 in wells infected with the highest viral dose, and even inoculation with a 10^3^ dilution of the virus resulted in a measurable OD signal (Fig. 3b). Because of plaque formation at day 2 in wells infected with the two highest viral doses, the ODs decreased (10^1^) or remained constant (10^2^), and spreading virus infection is likely the reason for the increase in OD values after inoculation with 10^3^ and 10^4^ dilutions of the virus (Fig. 3b). Thus, the antibody-HRP based technique is more sensitive and allows detection of ZIKV as soon as 24 h post-infection. However, detachment of virally infected cells represents a confounding factor that needs to be taken into consideration when performing antibody-based readouts. The MTT-based assay that relies on the virus-induced CPE allows quantification of viral infectivity at later time points and also over a broader range of viral inocula. This is a major advantage, because it allows analysis of multiple rounds of viral replication.

**Fig. 4:**
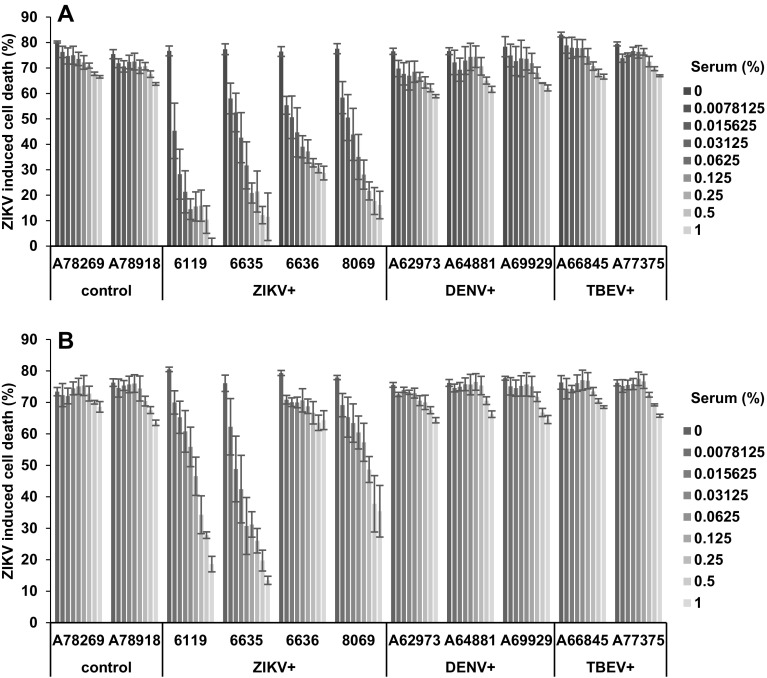
MTT assay allows quantification of the virus neutralization capacity of antisera from ZVD patients. Negative control sera or sera positive for ZIKV (ZIKV+), Dengue (DENV+), or tick-borne encephalitis virus (TBEV+) IgG and IgM antibodies (Table 1) were diluted and incubated for 90 min with 1 × 10^6^ TCID_50_/ml of the lab-adapted ZIKV strain MR766 (**a**) or the recent clinical isolate GWUH (**b**). Thereafter, 180 µl Vero E6 cells were infected with 20 µl of the virus/antisera samples, and 4 days later MTT assay was performed. Shown is the percentage of virus-induced dead cells obtained from triplicate infections ± standard deviation. Shown on the *x*-axis are the final cell culture concentrations of the sera in %

Having demonstrated that the MTT-based cell viability assay allows quantification of ZIKV infectivity, we next studied whether this assay could be adapted to determine the neutralizing antibody titers of well-characterized patient sera derived from confirmed Zika Virus Disease (ZVD) cases, and dengue virus (DENV) or tick-borne encephalitis virus (TBEV) infected individuals (Table 1). For this, 10^6^ TCID_50_/ml of the ZIKV MR766 strain were incubated with serial dilutions of inactivated serum samples for 90 min. Thereafter, 180 µl of Vero E6 cells were infected with 20 µl of ZIKV/antisera mixtures and the MTT-based cell viability assay was performed 4 days later. DENV and TBEV antisera modestly reduced the virus-induced CPE, but this effect is unlikely to be mediated by specific neutralizing antibodies as two control sera displayed a similar activity (Fig. 4a). Interestingly, the four sera from ZVD patients inhibited ZIKV-induced cell death in a dose-dependent manner (Fig. 4a, S4A). The most active serum was 6119 (Fig. 4a, S4A and B). Since the MTT assay yields values that can be processed electronically, we calculated the serum concentrations required to block ZIKV MR766 infection by 50% (IC_50_) or 80% (IC_80_) using the GraphPad Prism software. This analysis revealed that, e.g., 6119 inhibited ZIKV infection with an IC_50_ value of 0.013% and IC_80_ of 0.059% (Table 2). The IC_50_ values of the remaining ZIKV antisera were 0.043% (6635), 0.052% (8069), and 0.10% (6636) (Table 2). Similarly, plotting ZIKV-induced cell death against antiserum dilutions (Fig. S4) allowed to calculate the titers to block infection by 50 or 80%, respectively (Table 1). This analysis provides more exact quantitative values than those observed by classical readouts where only the last detected serum dilution is given (see IgM or IgG titers in Table 1) and where the titers are based on subjective plaque detection by eye (Table 1).

**Table 1:** Characteristics and ZIKV neutralization of analyzed patient sera

Serum	Designation	IgM (index or titer)	IgG (index or titer)	PRNT (titer)	MTT 50% inhibition		MTT 80% inhibition	
					MR766 (titer)	GWUH (titer)	MR766 (titer)	GWUH (titer)
Control	A78269	*0.1*	*0.3*	<20	<100	<100	<100	<100
Control	A78918	*0.04*	*0.4*	Nd	<100	<100	<100	<100
DENV	A62973	*0.6*	***6.1***	<20	<100	<100	<100	<100
DENV	A64881	*0.5*	***3.8***	<20	<100	<100	<100	<100
DENV	A69929	*0.03*	***4.2***	Nd	<100	<100	<100	<100
TBEV	A66845	*0.17*	***5.5***	<20	<100	<100	<100	<100
TBEV	A77375	Nd	***6.4***	Nd	<100	<100	<100	<100
ZIKV	6119	1280	2560	1280	**6719**	**554**	**1586**	<100
ZIKV	6635	2560	2560	3840	**2195**	**1600**	<100	**172**
ZIKV	6636	1280	320	960	**747**	<100	<100	<100
ZIKV	8069	1280	640	960	**1764**	**164**	**199**	<100

IgM and IgG antibodies against DENV and TBEV were determined by indirect ELISA and are shown as indices (*in italics*). IgM and IgG antibodies against ZIKV were determined by IIFT as described in material/methods section and give the titer (serum dilution) at which the fluorescence signal could last be detected. Control antisera were tested negative for anti-DENV IgM and IgG. The PRNT was performed with ZIKV MR766 and gives the titer (serum dilution) at which infected wells could be detected. The MTT-based assay allows calculation of titers (serum dilutions) at which ZIKV infection was blocked by 50 or 80%

**Table 2:** 50 and 80% inhibitory concentrations of ZKD sera against ZIKV

Antiserum	Designation	IC_50_		IC_80_	
		% serum in cell culture (v/v)			
		MR766	GWUH	MR766	GWUH
ZIKV	6119	0.013	0.172	0.059	>1
ZIKV	6635	0.043	0.056	0.410	1
ZIKV	6636	0.100	>1	>1	>1
ZIKV	8069	0.052	0.442	>1	>1

Values shown were calculated using GraphPad Prism

Thus, the MTT assay allows a rapid electronic determination of the neutralizing antibody titers (or inhibitory activities) without the need for a microscopic quantification of plaques performed in routinely used PRNT assays.

In all experiments, so far, we used the ZIKV strain MR766 that has been passaged in mice and cell culture multiple times [24]. We were wondering whether the MTT assay also allows analysis of a more recent clinical ZIKV isolate. For this, we used ZIKV FB-GWUH-2016 that was derived post-mortem from the brain of a fetus with birth defects [20]. This isolate (termed GWUH herein) was passaged two times on Vero E6 cells where it also causes plaque formation (data not shown). We determined neutralizing antibody titers of all antisera against the GWUH isolate under exactly the same conditions as for MR766. Again, we observed that control, DENV, and TBEV antisera slightly reduced plaque formation (Fig. 4b). Interestingly, only three of the four ZIKV antisera (6119, 6635, and 8069) suppressed plaque formation by GWUH, whereas 6636 was almost inactive (Fig. 4b, S4C and S4D). In general, the MTT-IC_50_ values against GWUH were slightly higher as compared to MR766 (Tables 1, 2, Fig. S4C and S4D). Taken together, our data show that the MTT-based cell viability assay not only allows quantification of ZIKV neutralizing antibody titers but may also detect differences in the antibody response against ZIKV lineages.

**Fig. 5:**
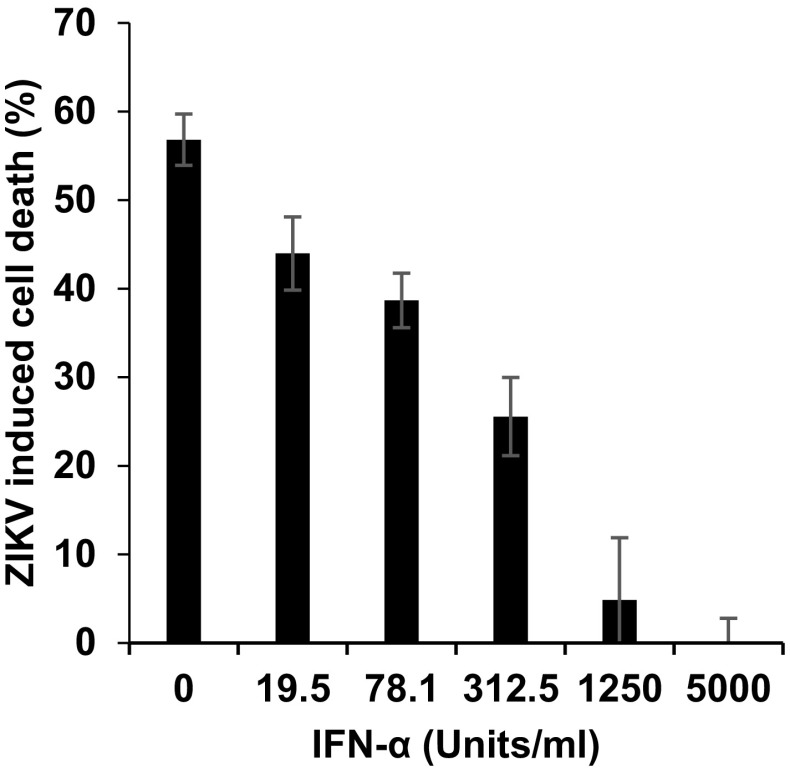
Interferon α inhibits ZIKV GWUH infection as shown by MTT assay. Vero E6 (170 µl) cells were incubated with 10 µl IFN-α at indicated concentrations for 2 h, before 20 µl ZIKV strain GWUH was added. 3 days later, MTT assay was performed. Shown is the percentage of virus-induced cell death obtained from triplicate infections ± standard deviation

Finally, we analyzed whether the MTT assay can be used to study inhibitors of ZIKV infection or replication. For this, Vero E6 cells were exposed to IFN-α which inhibits ZIKV infection [15], and were then inoculated with ZIKV GWUH. The MTT assay detected a dose-dependent suppression of ZIKV-induced cell death by IFN-α (Fig. 5). Thus, the MTT-based ZIKV detection assay allows studying antivirals and might be particularly useful for larger high-throughput screening approaches.

## Discussion

We describe, in this report, a reliable, robust, and cheap assay to determine ZIKV infectivity and its neutralization by antisera or suppression by antivirals. Our test is based on the ZIKV-induced cytopathic effects that result in cell death and cellular detachment which is indirectly quantified by measuring the metabolic activity of remaining adherent life cells. We show that the MTT-based viability assay has a very broad dynamic range (Fig. 1, S2 and S3) and an excellent Z’ factor. Moreover, MTT ODs (and hence, ZIKV -induced cell death) almost perfectly correlated with the viral inoculum (Fig. 1 and S2). At later time points, sigmoidal dose–response curves were obtained due to pronounced cell death caused by spreading infection in infected wells, whereas cells in uninfected wells remained alive (Fig. S2). However, ODs are derived from live (non-infected) cells. To define the percentage of cells that were killed by ZIKV, we calculated ΔOD values by subtracting ODs derived from uninfected cells from ODs from infected cells and normalized these data for viable cells in the untreated control. These values represent the percentage of cells that were killed by ZIKV and provide a more direct value for viral infectivity (Figs. 1, 2, and S3) or its inhibition by antisera (Fig. 4, S4) or interferon (Fig. 5).

The infectious titer of ZIKV is usually determined by TCID_50_ analysis using endpoint titrations and microscopic evaluation of the virus-induced CPE between 5 and 10 days post-inoculation [17, 28]. However, microscopy of virus-induced plaques requires experienced personnel and is laborious, in particular when a large number of samples have to be analyzed. Thus, we were wondering whether the MTT assay may provide an alternative methodology that allows for a more convenient analysis of infectious viral titers. For this, we have taken the data set obtained at day 8 post-infection (Fig. 1a) and calculated the TCID_50_ according to Reed and Munch [29]. Wells were defined as infected if the OD was decreased by more than three times of the standard deviation of uninfected wells. This resulted in the calculation of an MTT TCID_50_ of 1.2 × 10^7^/ml, which is in good accordance with the TCID_50_ obtained by microscopic plaque detection, which was 1.7 × 10^7^/ml [7]. Thus, the MTT assay allows fast and quantitative determination of the infectious ZIKV titer without the need for time-consuming microscopy.

Colorimetric quantification of virus-induced cell killing by MTT had been used before to determine titers of influenza virus [30], picornaviruses [31], or respiratory syncytial virus [32]. In addition, this assay has been adapted to measure the efficacy of antivirals against HIV-1 [33–35], or herpes viruses [36, 37] or to perform vaccinia virus neutralization tests (VNT) [38]. Since ZIKV neutralizing antibody titers have usually been determined by microscopy-based time-consuming PRNT, we were wondering whether the MTT-based VNT could provide an alternative. Our results show that the four antisera derived from acute ZVD cases dose-dependently suppressed cell killing allowing a convenient determination of half maximal effective concentrations (Fig. 4; Tables 1, 2). These antisera have previously been analyzed in the IIFA for anti-ZIKV-IgM and IgG. There seems to be a correlation between anti-ZIKV IgG titers with MTT EC_50_s measured in the VNT assays, but the small sample size does not allow a statistical meaningful analysis. However, the use of two different ZIKV strains in the MTT-based VNT resulted in different titers. This finding suggests that the assay may allow the serological discrimination of ZIKV African and Asian lineage-specific antibody responses, or to study possible differences in neutralization capacities to pathogenic versus less pathogenic ZIKV strains. Therefore, more ZIKV African and Asian lineage-specific antisera should be analyzed in the MTT-based VNT to further support these initial findings. In addition, cross-neutralizing flavivirus antibodies were frequently detected in PRNTs. In contrast, our MTT-based VNT was demonstrated to be highly specific and, thus, eliminates the need for parallel testing with other flaviviruses to exclude the serological cross-reactivities. Furthermore, the dose-dependent reduction of infection by the natural ZIKV inhibitor IFN-α (Fig. 5) suggests that the MTT assay can be applied for high-throughput screening for ZIKV inhibitors.

The prerequisite for the MTT-based detection of viral infection is the capacity of the virus to induce cell death. We have tested the cell culture adapted ZIKV strain MR766 and the clinical isolate GWUH, and found that both induce pronounced cytopathic effects in Vero E6 monolayers. Many other ZIKV isolates were reported to be cytopathic likely allowing their analyses by MTT assay [39–43]. We have established the protocol using Vero E6 cells, a kidney cell line derived from an African green monkey that exhibits some degree of contact inhibition after forming a monolayer. This is useful in growing slow replicating viruses or determining neutralization titers that are directed against the virus, but has some limitations, because the cells are not of human origin. For example, cellular receptors involved in ZIKV entry and replication may differ between African green monkey and human cells. However, ZIKV has also been described as cytopathogenic in human cell cultures [44–46]. In fact, our results show that ZIKV causes cellular detachment of HOS monolayers, and consequently, the MTT-based assay could be easily adapted to human cells (Fig. S3).

In general, every cell viability assay can be adapted to quantify virus-induced cell killing. For example, Zmurko et al. recently made use of the soluble MTS reagent [23] and Xu et al. applied a luminescence-based assay that measures intracellular ATP to study the effect of antivirals on ZIKV infection [47]. We here compared the later assay principle with the MTT assay, and obtained Z’ factors and signal-to-basal (S/B) ratios in the same dynamic range (Fig. 2). Thus, the MTT assay is a reasonable alternative to the more costly luminescence-based readout. Another cell viability assay that has been adapted to quantify virus-induced CPE is based on the reduction of resazurin dye to the fluorescent resorufin product in metabolically active cells. This assay has been developed to determine the infectious dose of influenza virus strains [48] and does not require solubilization of the product. Similarly, tetrazolium salts, such as XTT or MTS, have been developed that are reduced to water soluble formazan derivatives and do not require solubilization with organic solvents for absorbance measurement. This is in contrast to the insoluble formazan crystals that form in the MTT assay. This advantage of resazurin or XTT/MTS-based assays is, however, a disadvantage for our assay, because it would require an additional decontamination step to inactivate ZIKV. Addition of the DMSO/ethanol solution to solubilize formazan inactivates ZIKV [7] and thus allows measuring absorbance of microtiter plates without the risk of infection.

Finally, the key advantage of the MTT assay is its low prize. The results can be monitored directly by the naked eye (Fig. S1B), and quantification can easily be performed in normal plate readers that are far cheaper than luminometers or fluorimeters. In addition, the MTT salt itself is at least 50 times cheaper than XTT, MTS, or luminescence-based cell viability kits. We calculated the material costs for one MTT assay per well with ~2 cents, which corresponds to only ~US$2 per 96-well plate. Thus, the MTT assay is a promising alternative to determine ZIKV titers or its inhibition by antisera or antivirals, in particular for large sample numbers under resource-poor settings.

## Electronic supplementary material

Below is the link to the electronic supplementary material.

Supplementary material 1 (PDF 562 KB)
