# Supplementary material for: Development of a high-throughput colorimetric Zika virus infection assay
Source: Med Microbiol Immunol. 2017 Feb 7;206(2):175–85. doi: 10.1007/s00430-017-0493-2 (PMC5357303; doi:10.1007/s00430-017-0493-2)
Supplement: Supplementary file 1 — Supplementary material 1 (PDF 562 KB) [file 430_2017_493_MOESM1_ESM.pdf]

Supplemental material

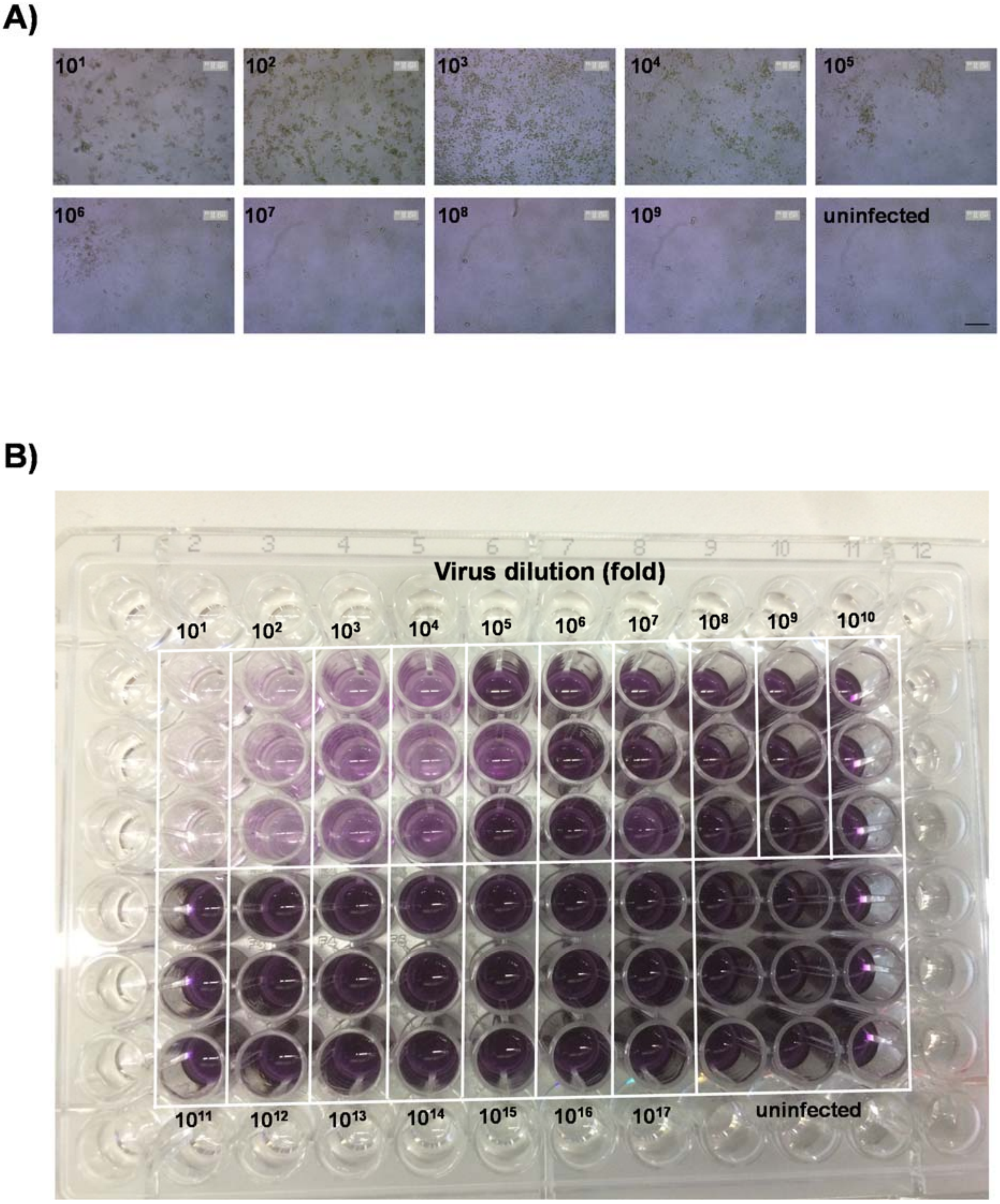

Figure S1. ZIKV induced cytopathic effect in Vero E6 cells and its quantification by MTT assay. 6 x 10^3 cells seeded in a 96 well plate were infected with serial 10-fold dilutions of ZIKV MR766 stock. **a)** At day 4, photographs of individual wells were taken showing cellular detachment and plaque formation after inoculation with 10^1 to 10^6 ZIKV dilutions. Scale bar is 200 µm. **b)** Photograph of the microtiter plate after dissolving the formazan crystals. At day 4, MTT reagent was added to the cells, after 3 hours of incubation the supernatants were removed, and the formed formazan crystals by live cells were dissolved in DMSO/ethanol.

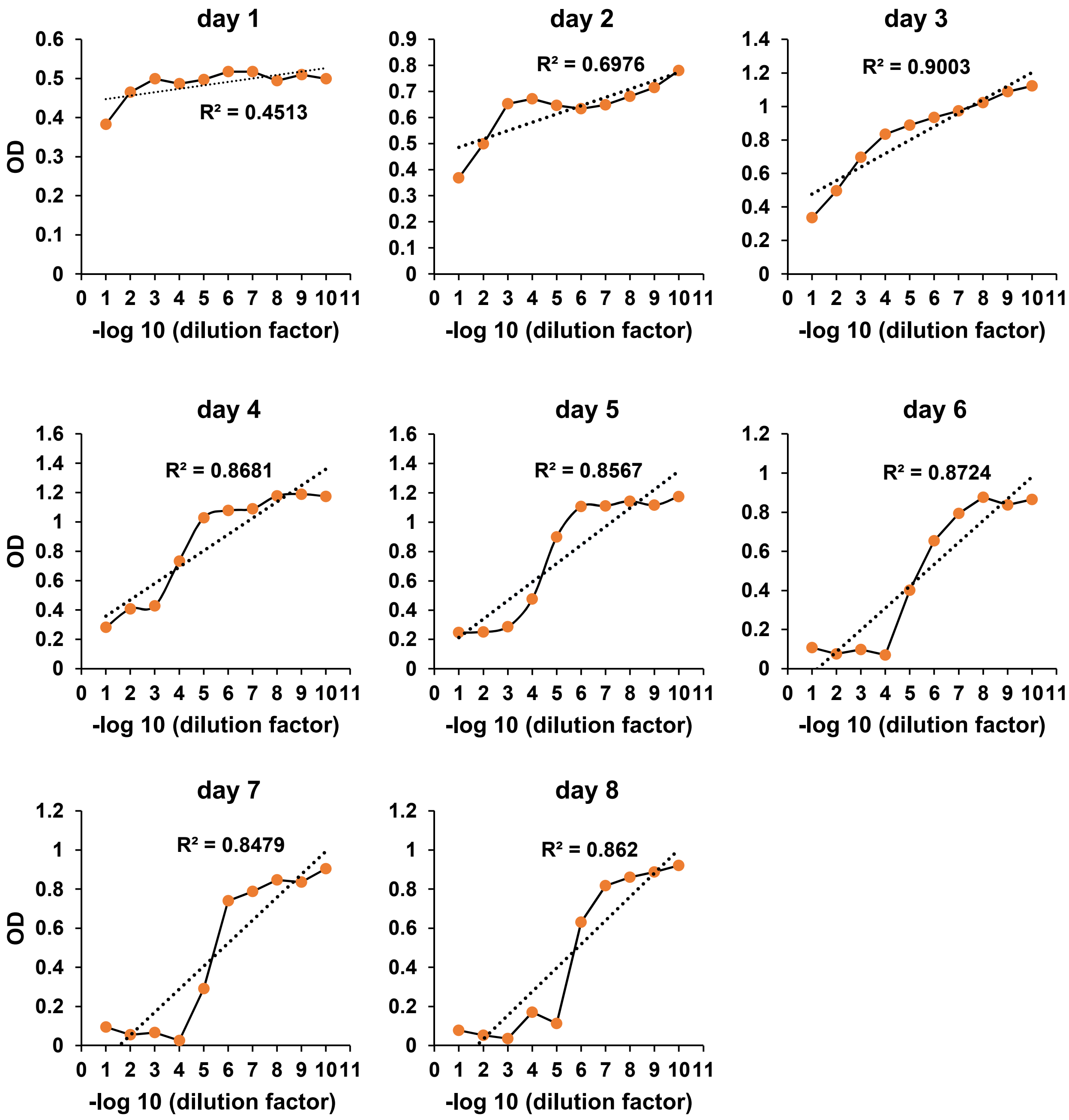

Figure S2. Correlation analysis of the ODs obtained after different times post infection with the viral inoculum. Linear regression analysis of data shown in Figure 1a. R^2 value is the coefficient of correlation.

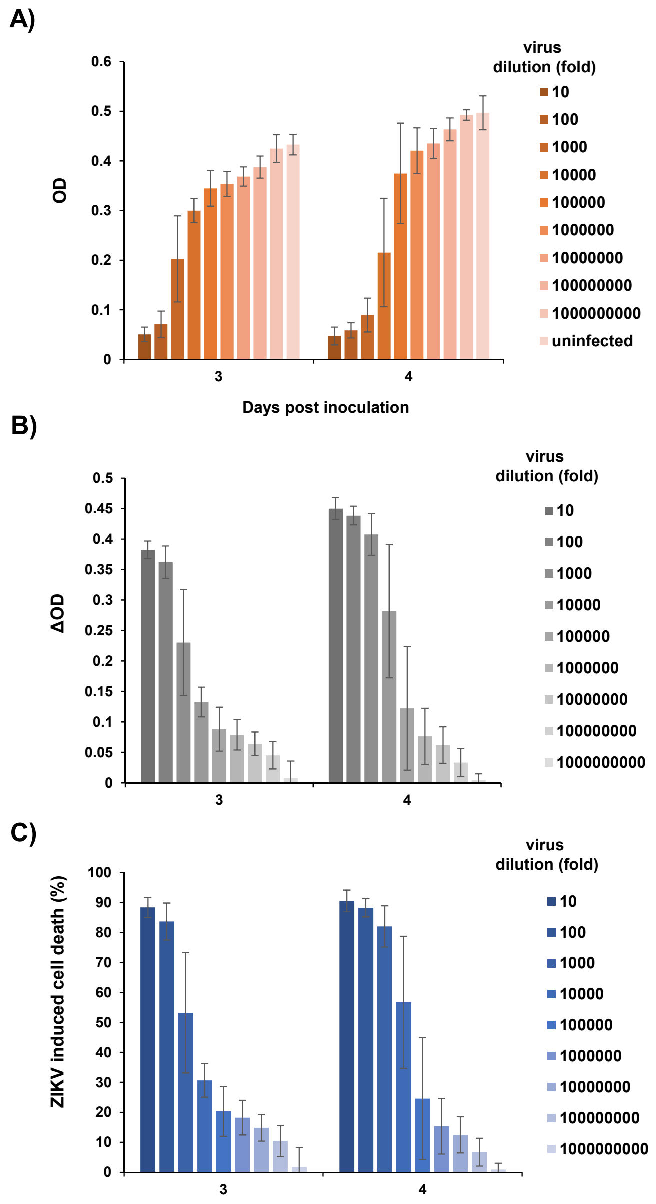

Figure S3. Representative results of the MTT-based assay to quantify ZIKV infection in HOS cells. 6 x 10^3 HOS cells were seeded in 96 well plates. The next day, 20 µl of serial 10-fold dilutions of the ZIKV strain MR766 were added. After 3 and 4 days, cells were monitored microscopically and washed once with medium to remove detached virally infected cells. Thereafter, 20 µl of MTT was added to remaining adherent cells in 200 µl. After 3 hours, formazan crystals were dissolved in DMSO/ethanol and absorbance of the colored solution was quantified by measuring absorbance at 490 nm by a spectrophotometer. **a)** Raw data obtained from live cells (optical densities; ODs) derived from triplicate infections ± standard deviation. **b)** ODs derived from live cells in infected cell culture were subtracted from ODs obtained from uninfected control cells. The resulting ΔODs numbers provide an indirect value for all cells that detached due to virus infection. **c)** The percentage of ZIKV induced cell death was calculated by normalizing the ΔOD values to the OD value of the uninfected control.

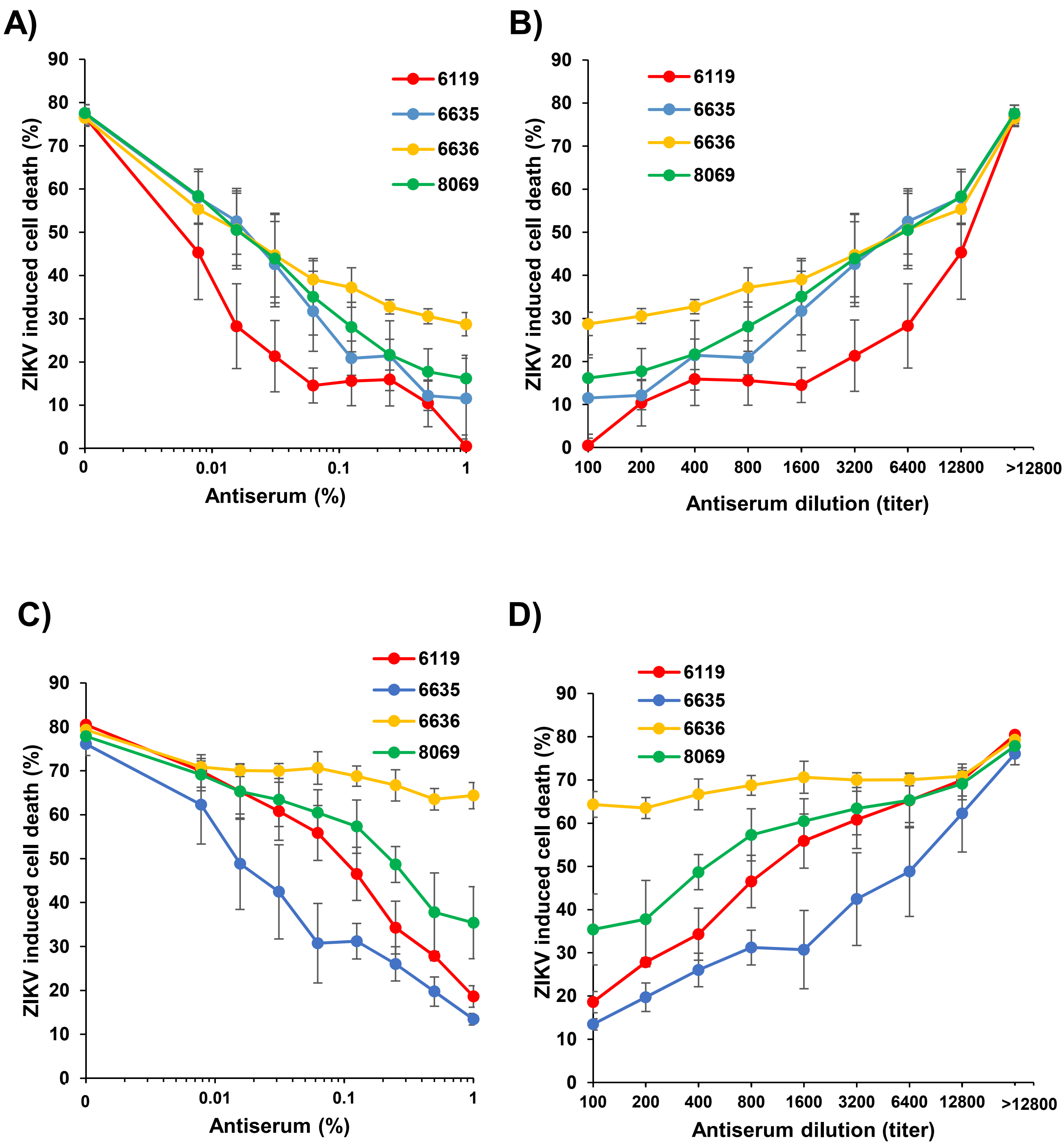

Figure S4. Neutralization of ZIKV infection by anti-ZIKV antisera. Dose-response curves of the four tested anti-Zika antisera against the MR766 (**a, b**) and the GWUH (**c, d**) strain. Values were obtained from the experiment shown in Fig. 4. In a) and c), the x-axis depicts the final concentration of antiserum in cell culture in % (v/v). In b) and d), the x-axis shows the antiserum dilutions (titer) allowing calculation of IC_{50} and IC_{80} values shown in Table 2.
